# Supplementary material for: Overcoming power-efficiency tradeoff in a micro heat engine by engineered system-bath interactions
Source: Nat Commun. 2023 Oct 27;14:6842. doi: 10.1038/s41467-023-42350-y (PMC10611737; doi:10.1038/s41467-023-42350-y)
Supplement: Supplementary file 1 — Supplementary Information [file 41467_2023_42350_MOESM1_ESM.pdf]

Supplementary information on Overcoming the power-efficiency tradeoff in a micro heat engine by engineered system-bath interactions

Sudeesh Krishnamurthy,[1] Rajesh Ganapathy,[2,3] and A. K. Sood[1,2]

[1] *Department of Physics, Indian Institute of Science, Bangalore - 560012, INDIA*

[2] *International Centre for Materials Science, Jawaharlal Nehru Centre for Advanced Scientific Research, Jakkur, Bangalore - 560064, INDIA*

[3] *Sheikh Saqr Laboratory, Jawaharlal Nehru Centre for Advanced Scientific Research, Jakkur, Bangalore - 560064, INDIA*

(Dated: October 8, 2023)

Table of contents:

1) Non-Markovian nature of Electrophoretic noise

2) Retreiving power efficiency tradeoff by increasing salt concentration

3) Controlling amplitude of input voltage along the isotherms

4) Averaging and Gaussian fits for $P(\Delta y)$ at low τ

5) Equilibration at $k_{\max}$ and $T_{\min}$

6) Calculation of work done and heat transferred

7) Comparison with previous experiments and theoretical studies

8) Ultimate fate of the heat engine and inspiration to engineering the regime of operation

9) Heat engine at higher temperature

Supplementary Note 1: Non-Markovian nature of Electrophoretic noise

Our scheme of attaining Carnot efficiency crucially depends on the nature of the noise used to drive the system. In the main paper, we utilized the electrophoretic force to generate such a noise and argued that the ballistic timescale τ_s depended on particle diameter and salt concentration. The origins of this force has been well studied and is part of reviews [1–3] for two decades. Here, we present the necessary formulae to support our claims.

A surface with a charge density q immersed in an aqueous solution attracts counterions and repels coions in its local proximity. The electrochemical double layer formed in the process screens the surface charge and creates an electrostatic potential, ϕ. In the Debye-Huckel limit of low surface charge, at a distance x from the surface,

$$\phi(x) = \frac{q}{\epsilon_w \kappa} e^{-\kappa x} = \zeta e^{-\kappa x} \tag{1}$$

where, ϵ_w is the permittivity of the solvent (DI water in our case, where $\epsilon_w = 80\epsilon_0$, permittivity of free space). ζ reflects the potential drop across the screening cloud and is commonly referred to as the zeta potential. κ is the inverse of the Debye screening length λ_D, given by

$$\lambda_\mathrm{D} = \sqrt{\frac{\epsilon_W k_\mathrm{B} T}{2 n_0 (ze)^2}} \tag{2}$$

for symmetric z:z electrolyte. Here, k_B is the Boltzmann constant, T the temperature, n_0 is the ion concentration in the solution, z the valency of the ions and e the ion charge.

Under the action of an electric field, electrochemical flows are induced in the vicinity of the trapped particle due to rearrangements in the double layer and they exert a force on the trapped particle. The underlying electrochemical mechanisms have been studied for the case of freely diffusing particles in an electric field for over the last two decades [1, 4, 5] and, the relaxation times of these flows can be $\approx 1\mu$s to 10 ms. The shortest relaxation times correspond to inertial relaxation [4], where flows dissipate over $\approx 1\mu s$. Frequency dependence of the dielectric constant of the solution results in gamma dispersion [1] and these flows decay over timescales $\approx 1\mu$s as well. Maxwell-Wagner relaxations or delta dispersion arise from difference in the dielectric constants of the trapped particle and surrounding solvent [1]. The resulting flows due to the delta dispersion decay over $\approx 0.01 - 0.1$ ms. Alpha relaxation occurs due to the difference in the nature of polarization of the counter and coions around the colloidal particle [1]. The ensuing flows relax in timescales $\approx a^2/D$,

where a is the particle radius and D, the ionic diffusion co-efficient. For the system used in our experiments, these relaxations occur in $\approx$ 13 ms. Finally, a global electrochemical flow occurs due to the charging of the electrochemical cell used in the experiment. The capacitance of our electrochemical cell is $\approx 7.8X10^{-11}$F when filled with a dielectric with the same permittivity as the electrolyte. With the resistance of the wires connecting our cell to the voltage source $\approx 94\mu\Omega$, the charging time would be $\approx 1.7X10^{-15}$s. In practice, the charges would be pumped to the electrodes in the timescale in which the voltage generator switches between voltages $\approx 1\mu$s $<< \tau_{\text{s}}$. Simultaneous to the rearrangement of charges around the bead, there would be a similar redistribution that occurs due to the ion build up near the electrodes [6]. This charging would occur on a timescale $\approx \frac{\lambda_{\text{D}} L}{D}$, where L is the distance between the electrodes of the cell. For the experimental cell used in our experiment, this timescale is $\approx$ 250ms $>> \tau_{\text{s}}$. Further, as pointed in the main paper, the particle motion in the optical trap occurs over a relaxation time $>$ 100ms. Thus, at the timescale of the rearrangement of ions around the colloidal particle, the changes in the charges on other surfaces involved in the experiment would be negligible. While multiple relaxation mechanisms have been discussed for freely diffusing particles, such models remain to be developed for optically trapped particles [7–10].

Phenomenological modeling of forces on an optically trapped bead due to an applied DC field (such as in Fig. 1b of the main paper) in previous studies [7] have revealed a triple exponential increase and decrease. At timescales $\approx 5 - 10$ms used in Fig. 1a, only the exponential saturation timescale is relevant. This simplistic case, historically referred to as the capacitor charging model, forms the origin of the non-Markovian noise in our experiment. The interactions between colloidal particles and an applied oscillating electric field are in general much more complicated and depends sharply on the frequency of the applied AC field. To verify if this model sufficiently describes the non-Markovian noise, we fit the observations in Fig. 1b of the main paper to the equation for the charging of a capacitor

$$\langle y \rangle = y_f - y_f e^{-t/\tau_{\text{s}}} \tag{3}$$

where y_f is the saturation value of $\langle y \rangle$. In Supplementary Figure 1a, we fit the average position of the particle to the charging equation. The corresponding equation for the fit is displayed in the panel and the measured $\tau_{\text{s}} = 5.28 \pm 0.44$ ms.

As a consequence of the deterministic nature of the charging process described in Supple-

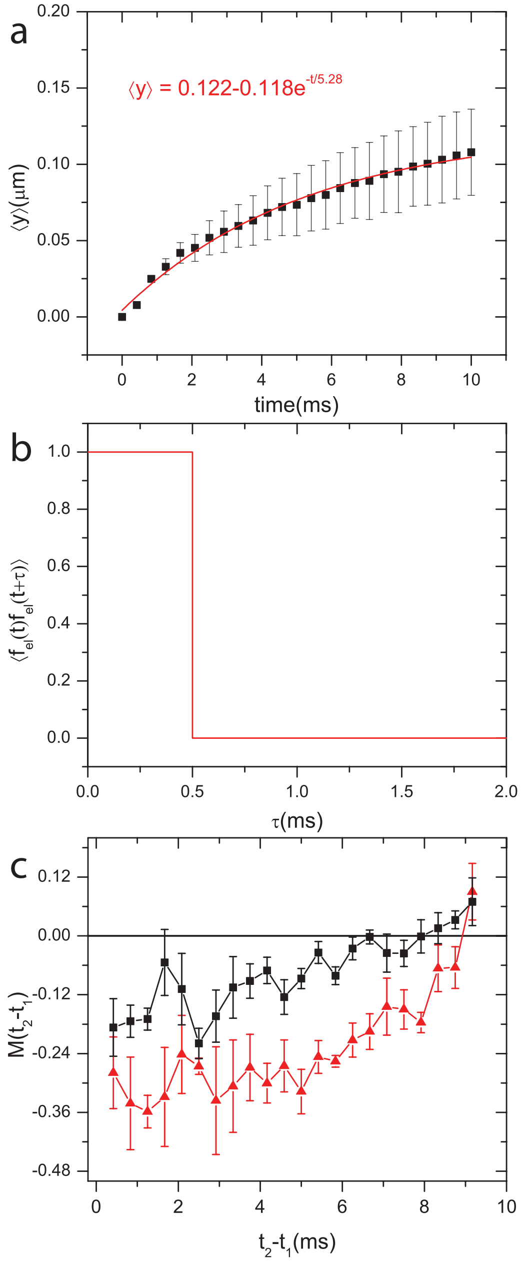

Supplementary Figure 1. **Particle motion during charging of the capacitor.** **a** shows capacitor charging model fits for $\langle y \rangle$ as a function of time. The red line represents the fit for the experimental data and its equation is displayed. **b** is the correlation between the electro-osmotic forces. The correlation is non-zero over the sampling interval signifying that the dynamics during this period would be deterministic. **c** is plot of $M(t_2 - t_1)$ for $t_3 = 9.58ms, t_2 = 9.16$ ms with $\Omega_1 = \Omega_2 = [-1, 0.5]$ for the data of $V_{\text{in}} = 0.5$V in Fig. 1b (red triangles) and $\Omega_1 = \Omega_2 = [-1, 2]$ for $V_{\text{in}} = 1.2$V in Fig. 1b (black squares). Averaging and error bars are the same as Fig. 1

mentary Figure 1a, the forces experienced by the particle, f_{el} are also deterministic over the sampling time of the noise in our experiment = 0.5 ms (Supplementary Figure 1b) signifying the non-Markovian nature of the noise. Thus, τ_{s} is the timescale over which the particle maintains its direction of motion as observed in Fig. 1b of the main paper and source of the memory in the electrophoretic noise in our experiment. To quantify the memory in the particle motion during the charging process, we calculate a memory function $M(t_1)$ defined by A. Seif et.al. [11].

$$M(t_1) = \frac{\langle x_{t_3} | x_{t_2} \in \Omega_2, x_{t_1} \in \Omega_1 \rangle}{\langle x_{t_3} | x_{t_2} \in \Omega_2 \rangle} - 1 \tag{4}$$

where $x_{t_1}, x_{t_1} \& x_{t_1}$ are particle positions at $t_1 < t_2 < t_3$ respectively and Ω_1, Ω_2 are subsets of particle positions. Intuitively, this would signify the influence of the motion during $t_2 - t_1$ on $t_3 - t_2$, which is independent for a Markovian process with $\langle x_{t_3} | x_{t_2} \in \Omega_2, x_{t_1} \in \Omega_1 \rangle = \langle x_{t_3} | x_{t_2} \in \Omega_2 \rangle$. Thus, $M(t_1) = 0, \forall t_1$ for a Markovian process and is non-zero otherwise. In general, $M(t_1)$ could be dependent on the choice of $\Omega_1, \Omega_2, t_2, t_3$. To calculate $M(t_1)$ for the charging process in our system, we follow A. Seif et.al. [11] and fix these parameters and change $t_2 - t_1$. The resulting $M(t_2 - t_1)$ is plotted in Supplementary Figure 1c. As anticipated from the deterministic nature of the electrophoretic forces, $M(t_2 - t_1)$ is indeed non-zero during the charging process and signifies the non-Markovian nature of the electrophoretic noise.

In the phenomenological model described in [7], τ_{s} can be set using the free parameters a, n_0 and z. In Fig. 3b of the main paper, we modulated a and showed that it allowed us to tweak system-bath interactions. In section II, we demonstrate the same using $n_0 z^2$.

To characterize the effects of the electrophoretic noise on particle dynamics, we plotted noise characteristics in Fig. 2 of main paper. Here, we present the experimental details involved in them. In Fig. 2a of the main paper, we plotted the probability distribution of displacements, $P(\Delta y)$ before and after applying the electrophoretic noise and demonstrated that it results in an increase in the width of $P(\Delta y)$. This would translate into an increase in T_{eff} only if the corner frequency of PSD_y remains constant during the process. To demonstrate this, we examined PSD_y before and after applying the electric field in Supplementary Figure 2a (Also Fig. 2b of the main paper). The solid lines correspond to Lorentzian fits to the data and the corner frequency before and after applying the field was observed to be 2.21 ± 0.3 Hz and 2.23 ± 0.4 Hz respectively. Thus, the corner frequency remains unaffected by the electrophoretic noise within the limits of experimental error. The corresponding value

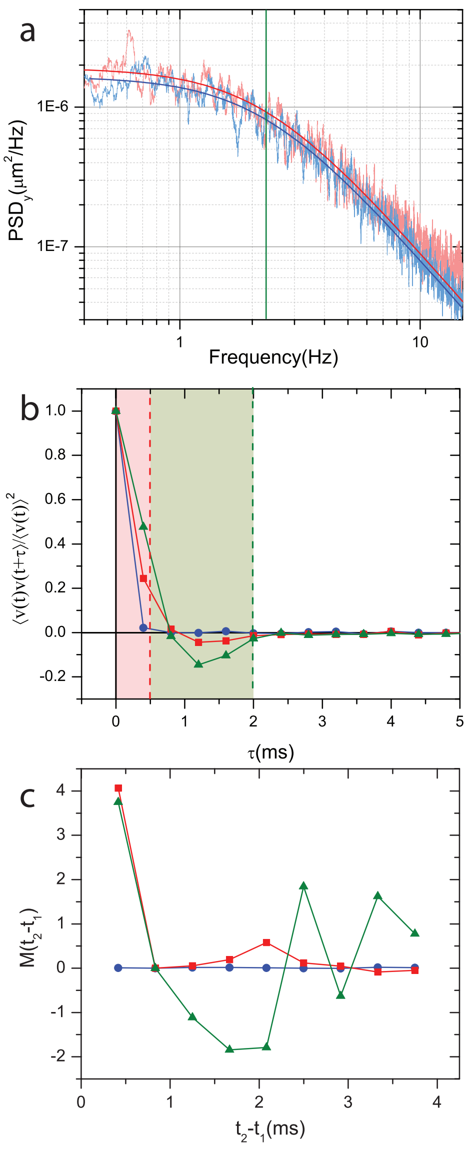

Supplementary Figure 2. **Noise characteristics.** **a** shows power spectral density of y displacements before (blue line) and after (red line) after applying the electric field. The solid lines represent Lorentzian fits for the data. **b** shows velocity autocorrelation function, VACF before (blue circles) and after applying the 2kHz (red squares) and 500Hz (green triangles) voltage noise. While the force is delta correlated before applying the noise, it is correlated over the red shaded region for 2kHz noise and red and green regions for the 500Hz noise. **c** is plot of $M(t_2 - t_1)$ for constant t_3 and t_2 with $\Omega_1 = \Omega_2 = [-0.1, 0.15]$ before (blue circles) and after applying the 2kHz (red squares) and 500Hz (green triangles). Averaging and error bars are the same as described in Fig. 2.

for the trap stiffness, k was 0.26 ± 0.05pN/μm and matched well with the measurements from the equipartition method of 0.32 ± 0.02pN/μm. The range of corner frequency used in our experiments presents a unique challenge in the measurement of the power spectrum, where one needs to obtain statistics at over a thousand frames per second for over thousands of seconds to reduce the experimental error. The data presented in Supplementary Figure 2a was measured at a sampling of 1kHz for over 200s. The spurious peaks in the red curve at low frequencies is a hallmark for the low statistics of the experiment and increase the error in determination of k. In order to circumvent this unnecessary challenge in our experiments, we calculated k by measuring $P(\Delta y)$ and used the equipartition theorem method.

The input voltage noise in our experiment was sampled at a frequency higher than the rate at which the particle position was measured. Given that the system operates in a low Reynold's number regime, such a fast switching of the electric field should ensure that the particle dynamics would remain uncorrelated on a timescale of position sampling. Such an uncorrelated noise would ensure that unlike the case of active fluctuations, the electric field would only add energy into the particle dynamics and not informational entropy. In order to verify this, we observed the velocity autocorrelation function, VACF of the trapped particle with and without the voltage noise in Supplementary Figure 2b (Also Fig. 2c of the main paper) by measuring the particle position at a high frame rate of 2500Hz. In the main text, we calculated that the ballistic timescale $\tau_{\rm b} \approx 10\mu$s. In Supplementary Figure 2b, however, the first time step of measurement is 400μs and the VACF decays to zero within this period for the particle in the absence of the applied field (blue line in Supplementary Figure 2b). For the 2 kHz noise, however, the applied force is deterministic for 500μs (the red region in Supplementary Figure 2b). This in turn is reflected in the particle dynamics, which now contains about 25% correlation at the first time step at 400μs and reduces to less than 10% in about 600μs. All experiments with $\tau_{\rm cycle} > 15$ ms were performed at frame rates where particle position was sampled beyond this timescale. To compare this with a noise with a larger sampling time, we performed a similar experiment with noise sampled at 500 Hz. The VACF of this noise is plotted in Supplementary Figure 2b (green line) and shows even larger correlation ($\approx 50\%$ at 400μs) that decays to zero in 2ms.

While longer relaxation time of the VACF is only an indicator of non-Markovian noise, the non-Markovianity of the noise can be quantified using the memory function discussed in Eqn. 4. In Supplementary Figure 2c (Also Fig. 2d of main paper), we calculate $M(t_2 - t_1)$

with $t_3 - t_2 = 0.416$ms with $\Omega_1 = \Omega_2 = [-0.1, 0.15]$. $M(t_2 - t_1) = 0$ in the absence of the applied field (blue circles in Supplementary Figure 2c) and is non-zero for the applied voltage noise at 2kHz (red squares in Supplementary Figure 2c) and at 500Hz (green triangles in Supplementary Figure 2c) and confirms that the electrophoretic noise experienced by the particle is non-Markovian in nature.

Supplementary Note 2: Retreiving power efficiency tradeoff by increasing salt concentration

In the main paper, we pointed that the system-bath interactions can be tuned by manipulating salt concentration in the suspending medium to retrieve the conventional power-efficiency tradeoff. In the previous section we argued that the ion concentration can be used to tweak τ_{s}, the ballistic timescale of the noise in the experiment. In this section, we present the consequences of decreasing τ_{s} by adding salt to the suspension and demonstrate that the power-efficiency tradeoff can be retrieved. To this extent, we present the corresponding results in Fig.s 3, 4 and 5 of the main paper for an engine operated with an elevated salt concentration in the solvent. Supplementary Figure 3a is equivalent to the relation between T_{eff} and k presented in Fig. 3b of the main paper. The exponent α of the logarithmic dependence decreases with increase in concentration of the salt $MgCl_2$ (Supplementary Figure 3a) and approaches the behavior observed for thermal noise ($\alpha = 0$). As a result, τ_{R} during the isochoric process, measured in Fig. 3d of the main paper for DI water to be 12 ms, increases to 30 ms (Supplementary Figure 3b) at a salt concentration of 700μmol $MgCl_2$. W_{C} and η obtained by operating a Stirling cycle described in Fig. 4 a and b of the main paper using such a solvent (700μmol $MgCl_2$) are plotted in Supplementary Figure 3, c and d. From Supplementary Figure 3c and d, while the $P - \eta$ tradeoff is temporarily violated in the range $\tau_{\mathrm{l}} > \tau > \tau_{\mathrm{h}}$, η eventually decreases as $\tau_{\mathrm{cycle}} \to 0$. In Fig. 5 of the main paper, we demonstrated that the engine with DI water as the suspending medium could violate the tradeoff for $\tau < \tau_{\mathrm{h}}$ as T_{eff} during the hot isotherm was higher than the cold isotherm and this was achieved using fast equilibration in the isochores. Since the isochores now equilibrate over longer τ_{R} (Supplementary Figure 3b), achieving such a cycle in the $k - T_{\mathrm{eff}}$ plane is not possible. Supplementary Figure 3, e and f are plots in the $k - T_{\mathrm{eff}}$ plane for the saline solvent similar to those in Fig. 5. For low $\tau_{\mathrm{cycle}} = 15$ ms (Supplementary Figure 3f) we see

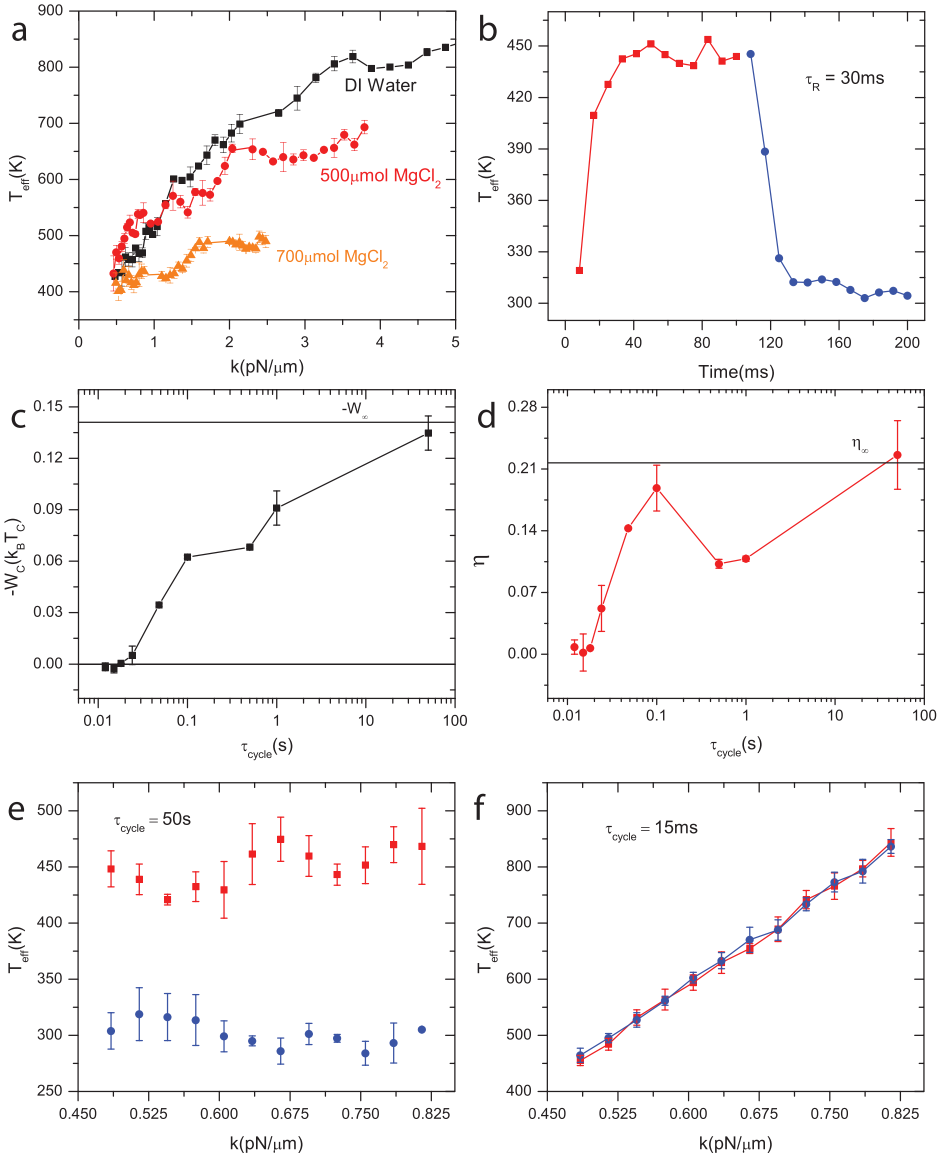

Supplementary Figure 3. Breakdown of reversal of $P-\eta$ tradeoff with salt concentration. **a** shows relationship between $T_{\rm eff}$ and k at elevated salt concentrations similar to Fig. 3b. Higher salt concentrations lead to a decrease in α and an increase in $\tau_{\rm R}$ during the isochoric processes as shown in **b** for 700μmol $MgCl_2$. **c** and **d** show $W_{\rm C}$ and η for the engine with 700μmol $MgCl_2$ as the suspending medium. While η shows an upturn in $\tau_{\rm l} > \tau > \tau_{\rm h}$ it eventually decreases to zero. **e** and **f** are plots of the state of the system in the $k - T_{\rm eff}$ plane for $\tau_{\rm cycle} = 50s$ and 15 ms. $T_{\rm eff}$ in the hot isotherm (red line) is no longer greater than the cold isotherm (blue line) at 15 ms. Averaging and error bars are the same as in Fig.s 3, 4 and 5 and is discussed in Supplementary Note 4

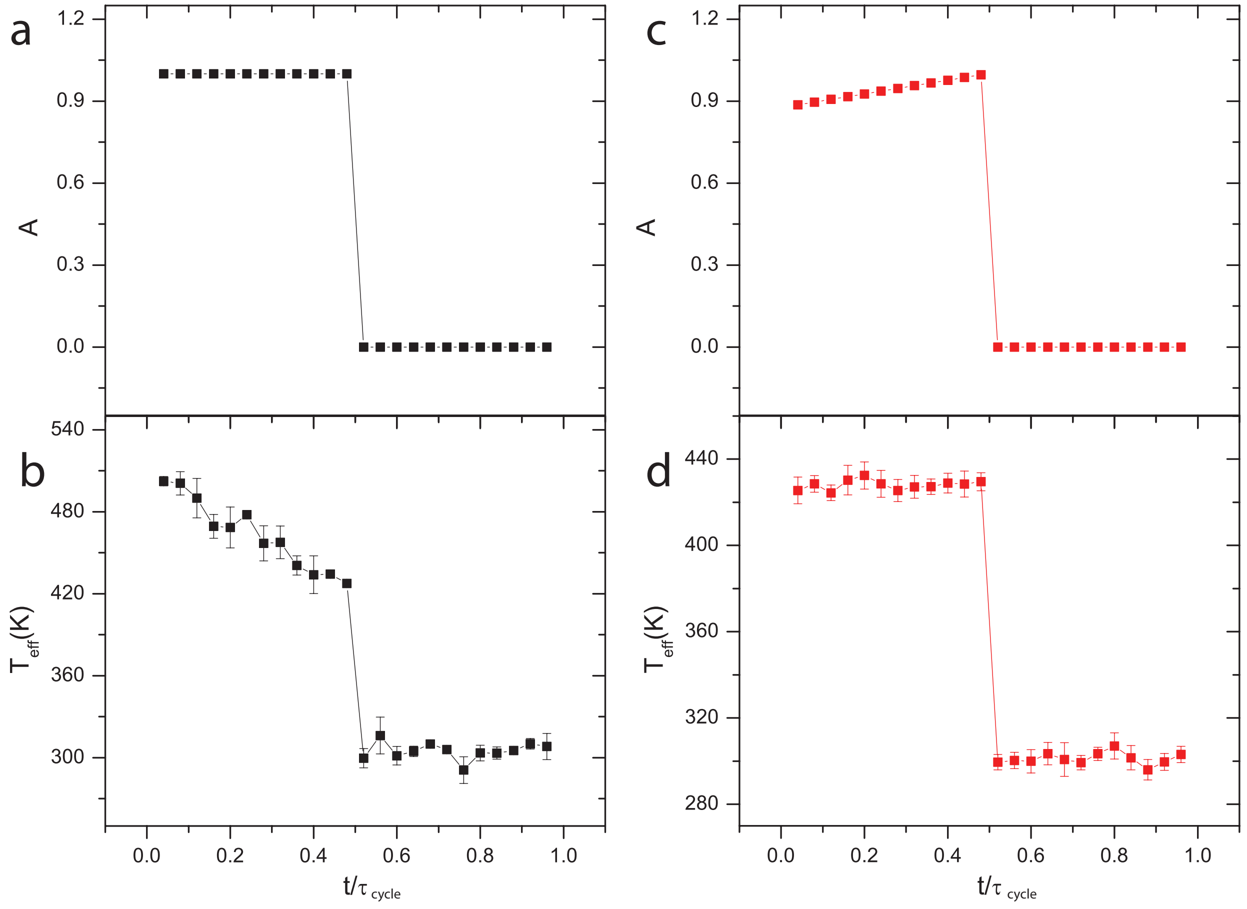

Supplementary Figure 4. **Controlling amplitude of input voltage along the isotherms** **a** is a typical modulation applied during the Stirling cycle if T_{eff} were independent of k. **b** shows T_{eff} along the cycle for the modulation in **a**. To correct for the change in T_{eff} along the hot isotherm, modulation in **c** was applied in our experiment. The resulting T_{eff} along the cycle is shown in **d**. Averaging and error bars are the same Fig. 3 and is described in Supplementary Note 4

that T_{eff} is almost same along both hot and cold isotherms and as a result, $P-\eta$ tradeoff is restored.

Supplementary Note 3: Controlling amplitude of input voltage along the isotherms

Executing the microscopic equivalent of Stirling cycle involves independently manipulating the trap stiffness, k and the effective temperature, T_{eff}. The control parameters used in our experiments to set k and T_{eff} are input laser power of the optical trap and voltage applied across the electrodes, V_{textin}. For the case of the non-Markovian noise, however, the

control parameters do not have a one to one correspondence with k and T_{eff} as noted in Fig. 3b of the main paper. While the trap stiffness k can still be adjusted using the input laser power, due to the dependence of k on T_{eff} (Fig. 3b of main paper), T_{eff} does not depend only on V_{textin} and this has to be accounted while performing the isotherms.

In the main paper, we described that we could maintain T_{eff} constant along the isotherms by modulating V_{textin}. In our experiments we induced such a modulation by electronically multiplying the input voltage by a modulation factor, A. Supplementary Figure 4a shows a typical modulation that would be applied to execute a Stirling cycle operating under a Markovian noise where there would be a one-on-one correspondence of T_{eff} with V_{textin} (1 during the hot isotherm ($0 < t/\tau_{\text{cycle}} < 0.5$) and 0 during the cold isotherm($0.5 < t/\tau_{\text{cycle}} < 1$)). The trap stiffness was simultaneously modulated using the input laser power as shown in Fig. 3a of the main manuscript. However, if this were to be applied in the range of k used in our experiment, T_{eff} decreases with k along the hot isotherms (Supplementary Figure 4b). To correct for the change in T_{eff}, we added the modulation in Supplementary Figure 4c. T_{eff} was constant in the resulting hot isotherm as shown in Supplementary Figure 4d and correspond to the microscopic equivalent of Stirling cycle discussed in Fig. 3a of the main text. We set the modulation corresponding to the largest cycle time of our experiment $\tau_{\text{cycle}} = 50s$, which was close to the quasistatic limit. The modulation was maintained constant across all τ_{cycle}.

Supplementary Note 4: Averaging, error estimation and Gaussian fits for $P(\Delta y)$ at low τ

Measurement of T_{eff} along the k and T_{eff} protocols in Fig.s 3 and 5 required averaging over large number of cycles. A straightforward approach is to perform an ensemble average over a very large number of cycles. This was indeed the strategy used to obtain Fig. 5d of the main paper. But such an approach would be limited by the frame rate used in our experiment and would invariably lead to smoothing of the data. On the other hand, at large $\tau_{\text{cycle}} = 50$ s, the number of cycles in our experiment was limited to only ≈ 200, which itself would amount to over 3 hours of data. Averaging over such small number of cycles would lead to very large error and the ensemble approach would be unviable. To work around this problem, we performed a binning average that maintained the same smoothing across all

$\tau_{\rm cycle}$ and allowed us to reduce the measurement error.

To achieve such an averaging, particle positions in each isotherm was isolated from the time series of measured positions and divided into 12 equal segments. Particle positions belonging to each segment were collected from several thousands of cycles and grouped. $P(\Delta x)$ corresponding to each group was individually plotted and $T_{\rm eff}$ was measured from the standard deviation of such distributions. Such an averaging yields 12 $T_{\rm eff}$ in each isotherm and are considered to correspond to the midpoint of the intervals. The procedure is repeated over several realizations of the experiment with a similar bead and the mean over these is plotted as $T_{\rm eff}$ in Fig.s 3 and 5 of the main paper. The error bars correspond to standard

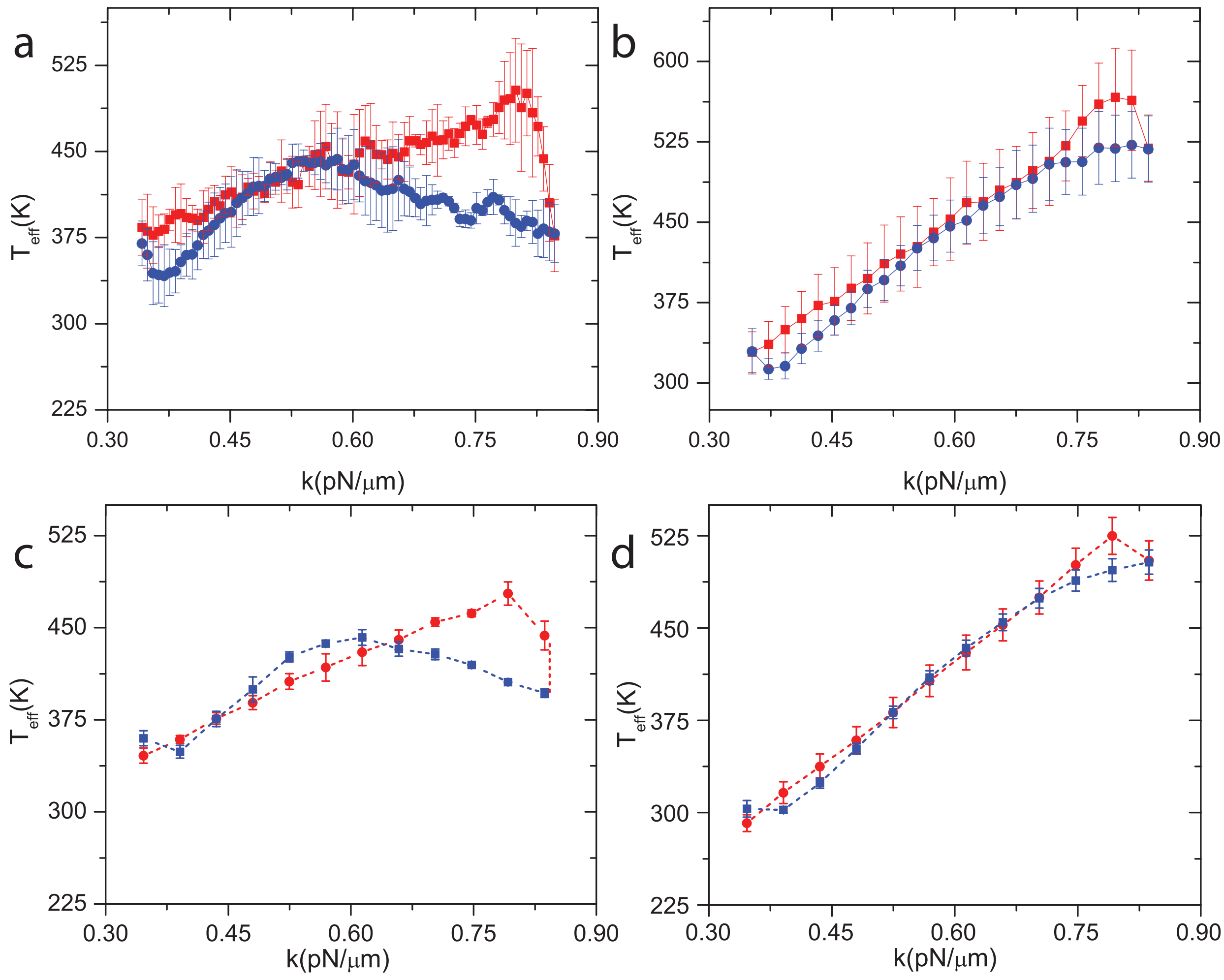

Supplementary Figure 5. **Comparison across different averaging strategies.** **a** and **b** show ensemble averaging for the data in Fig. 5b and Fig. 5c of the main paper (replotted in **c** and **d** for comparison). Error bars represent standard error of mean.

error of mean over these different realizations. Similar protocol was performed to obtain $\sqrt{\langle y^2 \rangle}$ in Fig. 3c of the main paper.

To compare across the averaging due to different strategies, we plot the data in Fig. 5b and 5c for $\tau_{\text{cycle}} = 300$ ms and 100 ms respectively in Supplementary Figure 5a and b with ensemble averaging. Fig. 5b and 5c are replotted in Supplementary Figure 5c and d for comparison. While the mean trajectory of the system in the $k - T_{\text{eff}}$ plane remains the same, the experimental error increases drastically with ensemble averaging. Details such as difference between T_{eff} of hot and cold isotherms at intermediate $k \approx 0.6 pN/\mu m$ are not clear due to such an averaging. The loss in information is more exacerbated at $\tau_c ycle = 50s$, where the error is up to $200K$. Since the plots in Fig.s 3 and 5 of the paper do not affect our central result of overcoming the power-efficiency tradeoff and are mainly to visualize the underlying processes, we used the binning average method.

The experiments in Fig. 4 were performed over four decades in time and experimental limitations allowed for different levels of averaging for each data point. The results for the longest cycle time $\tau_{\text{cycle}} = 50$ s were obtained from 11 independent realizations of the engine with 18 cycles each, amounting to a total of 198 cycles. The data point represents the average over the cycles and the error bars represent the standard error of the mean. The results of other cycle times were similarly obtained over multiple realizations of the experiment with the total number of cycles for $\tau_{\text{cycle}} = 1$ s amounting to 8100 cycles, $\tau_{\text{cycle}} = 500$ ms to 16000 cycles, $\tau_{\text{cycle}} = 300$ ms to 27000 cycles, $\tau_{\text{cycle}} = 100$ ms to 81000 cycles, $\tau_{\text{cycle}} = 48$ ms to 160000 cycles, $\tau_{\text{cycle}} = 24$ ms to 320000 cycles, $\tau_{\text{cycle}} = 20$ ms to 255000 cycles, $\tau_{\text{cycle}} = 18$ ms to 360000 cycles, $\tau_{\text{cycle}} = 16$ ms to 400000, $\tau_{\text{cycle}} = 15$ ms to 540000 cycles and $\tau_{\text{cycle}} = 12$ ms to 700000 cycles. Major contributions to the error bars arose from the variability between experimental realizations than due to the number of cycles performed at each τ_{cycle}.

In the main paper, we claimed that the state of the system could be represented in the $k - T_{\text{eff}}$ plane (Fig. 5 of the main paper) as the probability distributions, $P(\Delta y)$ fitted well with the Gaussian distribution. In Supplementary Figure 6, we illustrate this for eight state points sampled at different parts of the cycle for $T_{\max} = 427$ K at $\tau = 15$ ms explored in our experiment, where, violations, if any, should be detected. The solid lines represent Gaussian fits and, as can be seen from the figure, follow the experimental $P(\Delta y)$ within the limits of measurement error. Thus, T_{eff} can be defined using equipartition theorem.

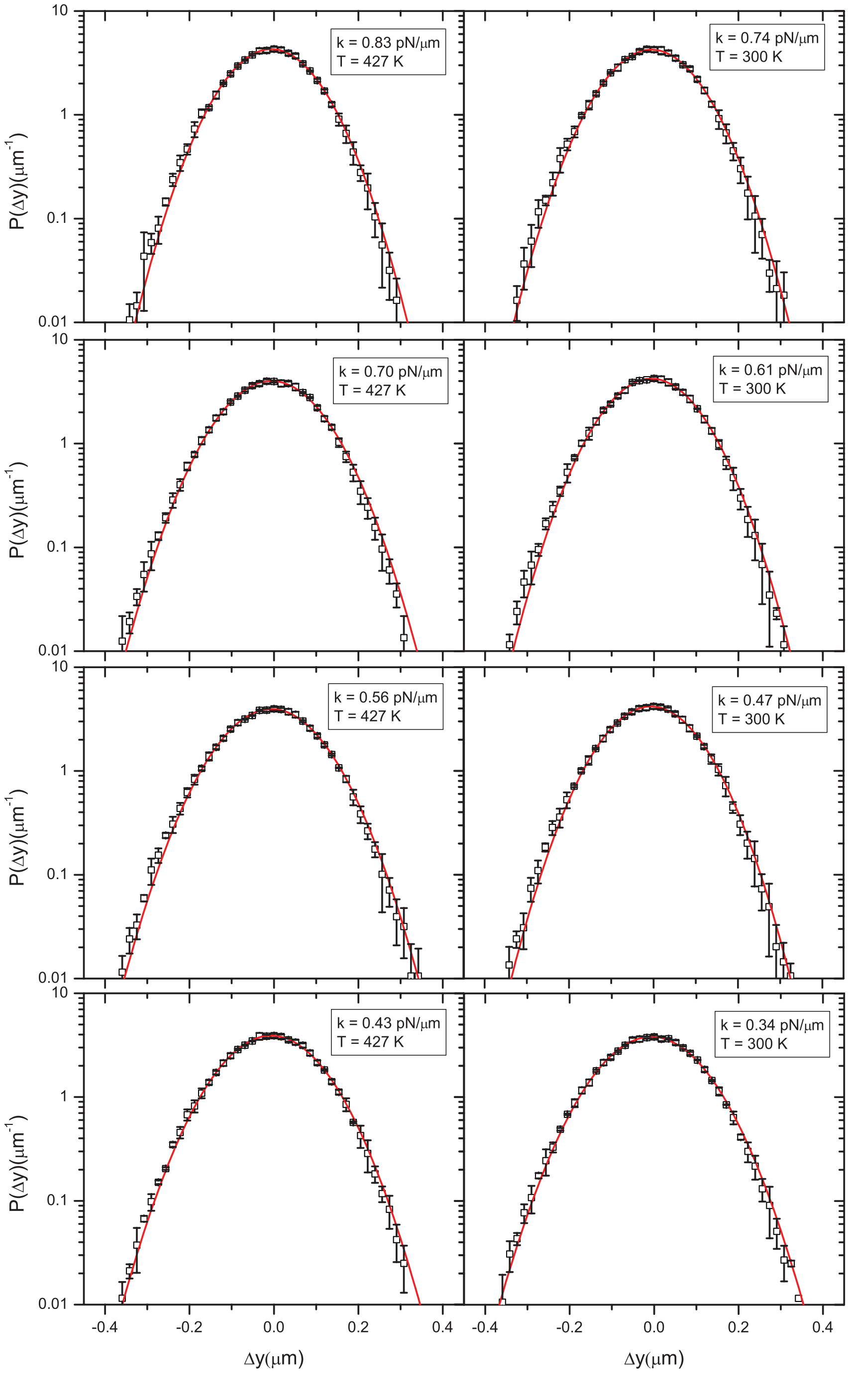

Supplementary Figure 6. **Gaussian profile of** $P(\Delta y)$ **at very low** $\tau_{\mathbf{cycle}}$**.** $P(\Delta y)$ at eight state points, denoted by their k and T_{eff}, for $T_{\max} = 427K$ at the lowest $\tau_{\text{cycle}} = 15$ ms are shown as black squares in the figure. The Gaussian fits for the same are shown as solid red lines in the figure. Averaging was performed over three different realizations of 540000 each and error bars represent standard error of mean over the realizations.

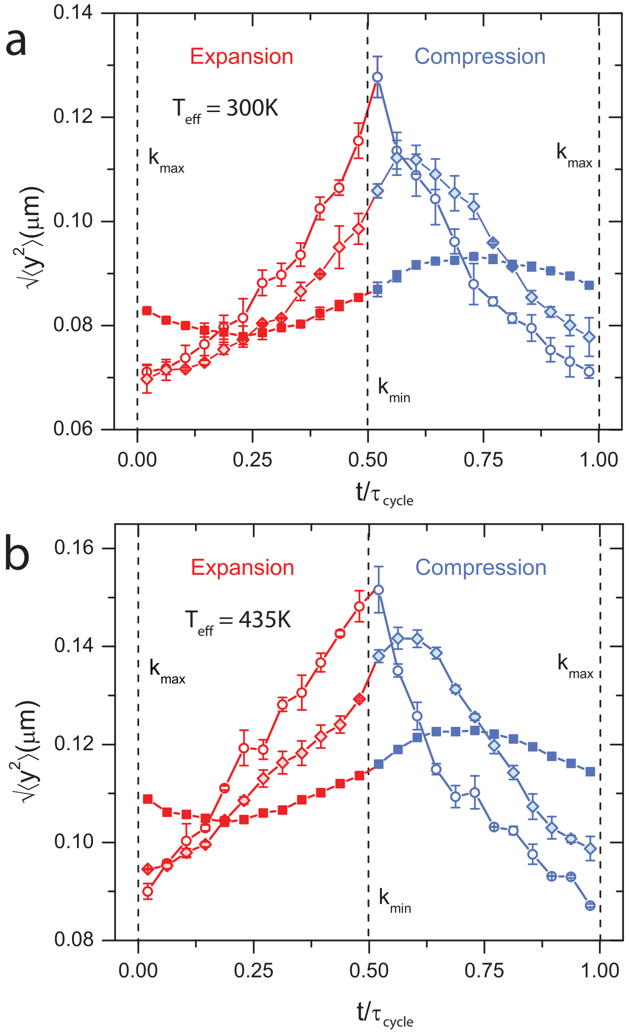

Supplementary Figure 7. Comparison of equilibration between the hot and cold isotherms. **a** is a plot of $\sqrt{\langle y^2 \rangle}$, the equivalent of volume in our engine along rescaled time $t/\tau_{\rm cycle}$ during the isothermal processes, where only the k protocol is performed at $T_{\rm eff} = 300$ K for $\tau_{\rm cycle} = 5$ s(open circles), 500 ms(triangles) and 100 ms(filled squares). **b** is a similar plot for the experiment performed for $T_{\rm eff} = 435$ K (also Fig. 3c). Comparison between **a** and **b** shows that $\tau_{\rm R}$ is the same across both isotherms. Averaging and error bars are the same as Fig. 3c and is presented in Supplementary Note 4

Supplementary Note 5: Equilibration at $k_{\rm max}$ and $T_{\rm min}$

In Fig. 3 c and d of the main paper, we determined τ_R during the isothermal and isochoric processes. However, in Fig. 3c, this was performed only at $T_{\rm max}$. In Supplementary Figure 7, we present a comparison of these results with a similar experiment performed at

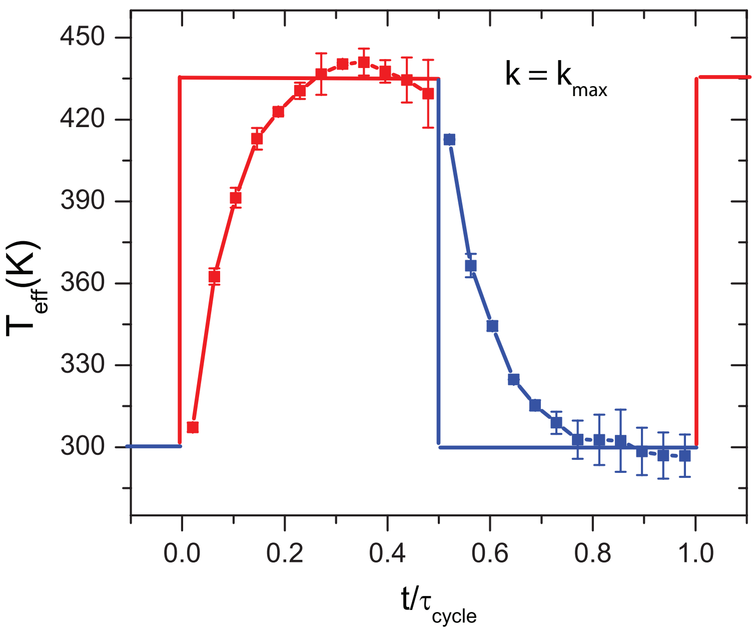

Supplementary Figure 8. **Thermal equilibration at** $k = k_{\mathbf{max}}$**.** Equilibration pathways for changes in T_{eff} during the isochoric process is plotted along t/τ_{cycle}, where only the T_{eff} protocol is performed at $k = k_{\text{max}}$ for $\tau_{\text{cycle}} = 50$ ms(closed squares). The particle equilibrates to the final value in $\tau_{\text{R}} = 11$ ms during both heating and cooling (as seen from data for 50 ms) and is significantly lower than 117 ms due to thermal noise.Averaging and error bars are the same as Fig. 3d and is presented in Supplementary Note 4

$T_{\text{min}} = 300K$. $T_{\text{min}} = 300K$ (Supplementary Figure 7a) was performed in the absence of electrophoretic noise and is similar to the results in the presence of such a noise (Supplementary Figure 7b). Thus despite system bath interactions, τ_{R} remained unaffected during the isotherms of our experiment due to the noise modulation.

Similarly, in Fig. 3d of the main paper, τ_{R} was determined only at $k = k_{\text{min}}$. In Supplementary Figure 8, we perform similar experiments at $k = k_{\text{max}}$. We observe that $\tau_{\text{R}} = 11$ ms during both isochoric heating and cooling. Thus, during our experiments, system bath interactions led to the decrease of τ_{R} in both the isochores performed at k_{max} and k_{min} .

Supplementary Note 6: Calculation of work done and heat transferred

In the main paper, we used the framework of Stochastic Thermodynamics [12, 13] to determine the work done and heat transferred during various cycles of the experiment.

Here, we present the formulae that were used in our analysis. Work done is obtained by evaluating the integral

$$W = \int \frac{\partial U}{\partial t} dt \tag{5}$$

Since the optical potential created by the trap is harmonic,

$$U = \frac{1}{2} k y^2 \tag{6}$$

where, k is the trap stiffness. Work done is obtained by discretization of the integral in (5) using (6)

$$W = \frac{1}{\nu} \sum_{0}^{t} \frac{1}{2} \dot{k}_y y^2 \tag{7}$$

where, ν is the frequency at which the position of the particle is measured. From (7) it is clear that work done is finite during Isothermal processes ($\dot{k}$ = constant) and zero during Isochoric processes ($\dot{k} = 0$).

Heat transferred is determined by evaluating the integral,

$$Q = -\int \frac{\partial U}{\partial t} \dot{y} dt = -\int k y \dot{y} dt \tag{8}$$

During the isothermal processes of the Stirling cycle, the trap stiffness is varied linearly and we have for isothermal expansion,

$$k = \frac{\Delta k}{\Delta t} t + k_{\text{min}} \tag{9}$$

Substituting into the integral in (8) and by integration by parts, we obtain heat transferred in the y degree of freedom, Q as

$$Q = -\left[k_{\text{max}} y(t)^2 - k_{\text{min}} y(0)^2 \right] + \frac{1}{2} \int_0^t \frac{\Delta k}{\Delta t} y^2 dt \tag{10}$$

For an equilibrium system, the first term in the RHS of (10) is the change in internal energy. For a isothermal process in the quasistatic limit, this term is zero. The second term is the work done by the engine in isothermal process. Thus, for the isothermal processes, heat transferred is equal to the work done. At low $\tau_{\text{cycle}} < 100$ ms, however, as T_{eff} decreases during the isotherm, this term contributes positive heat to the system and work is largely done at the cost of internal energy. Discretizing the integral in the second term, in a manner similar to equation (7) can be done only in the limit where the trap stiffness is varied on a timescale larger than the sampling time for measuring particle positions [14]. In our

experiments, at the lowest $\tau_{\text{cycle}} = 12$ ms, the particle positions were sampled every 0.5 ms. We maintained that a maximum of $1/12^{th}$ of the isotherm was performed during this sampling interval. The change in k during this interval was significantly lower than all colloidal engines designed hitherto [15]. The maximum frame rate at which the particle position could be sampled was limited by our apparatus at 2 kHz. Ensuring that at least $1/12^{th}$ of the isotherm when sampling at such a frame rate fixed the minimum $\tau_{\text{cycle}} = 12$ ms.

During an isochoric processes, the trap stiffness is constant and heat transferred can be calculated by evaluating the integral in (8).

$$Q = -k\int_0^t y\dot{y}dt = -\frac{k}{2}\int_0^t \frac{\partial y^2}{\partial t}dt \tag{11}$$

Evaluating 11,

$$Q = -\frac{k}{2}\left[y^2\right]_0^t \tag{12}$$

From equation (12), it is clear that the heat transferred during the isochoric processes depends only on the end points of the process.

There are three possible caveats that we wish to point out here. First, the efficiency in our experiment is the thermodynamic efficiency and not the fuel efficiency and is calculated from the heat transferred to the engine by the hot reservoir. This does not include the heat involved in maintaining the temperature of the reservoir. For example, the efficiency of a steam engine is calculated from the heat transferred to the engine and not from the coal burnt to heat the boiler. This has also been the historical definition of efficiency in equilibrium thermodynamics and stochastic thermodynamics. Second, whether the heat engine extracts only 'heat' from the reservoir or repackages 'heat' extracted from correlations is in general unclear. Recent studies have provided methods to resolve this by quantifying the heat extracted from such informational excess entropy [16]. The informational excess entropy can be calculated as

$$I = -\int dt P_y(t)\log\frac{P_y^{\text{S}}(t)}{P_y^{\text{eq}}(t)} \tag{13}$$

where $P_y(t)$ is the probability distribution of the system, $P_y^{\text{S}}(t)$ is the steady state distribution of the system and $P_y^{\text{eq}}(t)$ is the distribution of the system in the absence of external noise. In the case of all engineered noise, various mechanisms such as moving the optical trap [17], shining an exciting laser [18] and electrophoretic noise in [15] and our work, essentially a

driving force performs work on the system. Since the resulting noise experienced by the particle is similar to a system in equilibrium with an effective temperature, T_{eff} we expect that such work performed by the driving force would correspond to heat transfer from an effective reservoir. The choice of $P_y^{\mathrm{eq}}(t)$ as an equilibrium distribution devoid of any external engineered driving noise picks the work done by the driving force in changing the system to that at T_{eff} as information work. Although, this would technically be in line with the original definition [16], all heat transfers in the engineered reservoirs would just be work done. If however, $P_y^{\mathrm{eq}}(t)$ is chosen as the equilibrium reservoir at T_{eff}, it would then pick the work done by correlations in the input driving noise and this would be the intended purpose of calculating I. In our case, this would mean that $P_y^{\mathrm{S}}(t) = P_y^{\mathrm{eq}}(t)$ and $I = 0$. Thus, the noise used in our experiments is non-Markovian in the range of operation, but not an active noise. Again, the conventional definition of entropy would not neglect this term and considers it as the heat supplied by the reservoir even if the reservoir is out of equilibrium. Finally, the definitions of heat and work derived above, assume that the noise follows Markovian statistics. However, the input noise in our system is itself non-Markovian. There is however a general consensus that extension of the definitions is possible if the system is eventually Markovian at a larger timescale. This is indeed true in our system as noted in Supplementary Figure 2. It is in this sense that the definitions derived for Markovian systems are maintained in our experiments.

Supplementary Note 7: Comparison with previous experiments and theoretical studies

Experiments similar to the ones described in the main paper were performed earlier, but as pointed out, without considering the underlying relaxation times of the system. In their investigations into extending the Carnot limit to microscopic engines, Martinez. et.al. [15] depicted $P - \eta$ curves for a Brownian Carnot engine. The bounds used in these studies were $(k_{\mathrm{max}}, k_{\mathrm{min}}) = (20, 2)$pN/$\mu$m and $(T_{\mathrm{max}}, T_{\mathrm{min}}) = (525{,}300)$K with a colloidal bead of diameter, 1μ m. As described in Fig. 3b of the main paper, system-bath interactions would play an insignificant role at such high k and low particle diameter. $\tau_{\mathrm{h}} = 2.4$ ms in these circumstances were again smaller than the least $\tau_{\mathrm{cycle}} = 10$ ms examined. Also, only one τ_{cycle} was studied below $\tau_{\mathrm{l}} = 23.7$ ms. Hence, as anticipated by our results, only the initial decrease in W_{C}, η would be observed and can be verified from Fig. 2 of their paper.

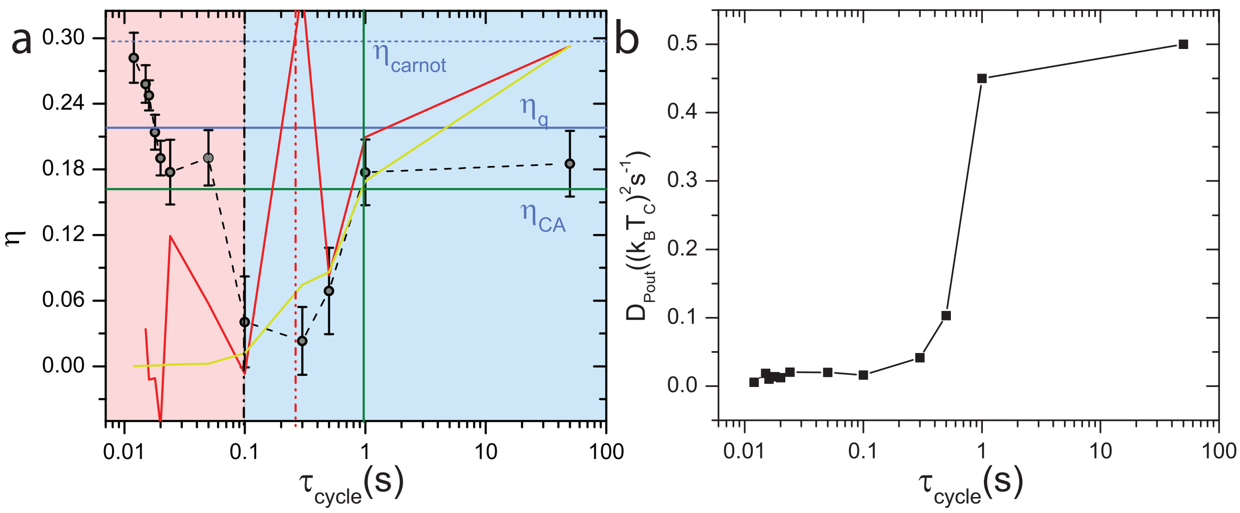

Supplementary Figure 9. **Comparison with theoretical predictions on efficiency at maximum power. a** shows efficiency of the engine for various τ_{cycle} (also presented in Fig.4b of main paper). The green lines mark the maximum in P in the range $\tau_{\text{cycle}} > \tau_{\text{h}}$ calculated in Fig.4c of the paper. η^* can be estimated from lines drawn on the plot of measured efficiency in **a**. The yellow line in **a** represents the bounds on efficiency proposed by Pietzonka and Seifert [21] and the red line that of Koyuk and Seifert [22] for thermal engines. Both the lines are greater than the measured efficiency for $\tau_{\text{cycle}} > \tau_{\text{h}}$. **b** represents power fluctuations with τ_{cycle} used to measure these bounds.Averaging and error bars are the same as Fig. 4 and is presented in Supplementary Note 4

In the absence of engines that could overcome the power-efficiency tradeoff, theoretical studies in the past have tried to predict at least the efficiency at maximum power, η^* - a key parameter in engine optimization. In Supplementary Figure 9 we compare our observations with these predictions. From the Fig. 4c of the main paper, had there been no reversal of the tradeoff for $\tau_{\text{cycle}} < \tau_{\text{h}}$, maximum power would be attained by the engine at $\tau_{\text{cycle}} \approx 1s$. The corresponding efficiency observed in our experiment was $\eta^* = 0.17 \pm 0.03$ (Supplementary Figure 9a). In their seminal work on engine optimization, Curzon and Ahlborn [19] predicted that $\eta^* = 1 - \sqrt{\frac{T_{\text{C}}}{T_{\text{H}}}}$, which has since been known as Curzon-Ahlborn efficiency, η_{CA}. For the parameters used in our experiment, $\eta_{\text{CA}} = 0.162$ and matches with the observed η^*. While η_{CA} has since been observed in a large class of experiments, it is neither exact nor universally observed in all heat engines. Revisions to this estimate was made by Schmeidel and Seifert [20] in their predictions on mesoscopic heat engines as $\eta^* = \frac{\eta_{\text{C}}}{2 - \eta_{\text{C}}/2}$. For the parameters in

our experiment, the predicted $\eta*$ from this work was 0.161 and also matched well with our observations. In a more recent study to establish an universal tradeoff relation, Pietzonka and Seifert [21] derived a novel tradeoff relation for thermal heat engines that proposed a bound on efficiency as $\eta \leq \frac{\eta_C}{1+2PT_C/\Delta_P}$. The yellow line in Supplementary Figure 9a represents this bound on efficiency calculated using the measured power, P and the power fluctuations, Δ_P and matches well with our data in the region $\tau_{\text{cycle}} > \tau_{\text{h}}$. The version of this bound corresponding to cyclic heat engines was derived by [22] as $\eta \leq \eta_C[1 + \frac{[P-\Omega\acute{P}]^2}{[\beta_C D_{Pout}P]}]^{-1}$ and corresponds to the red line in Supplementary Figure 9a. The large peaks in the predicted efficiency correspond to regions where $\acute{P}$ was zero and is a hallmark of the turnover in the behavior of the tradeoff. The predictions are again true only in the region $\tau_{\text{cycle}} > \tau_{\text{h}}$. It is to be noted that all these predictions were derived under the assumption that the reservoirs are Markovian. In practice, however, due to the finite nature of experimental reservoirs, particle and thermal currents are routinely generated, particularly at low cycle times. Given the practical scenarios, the assumption that the reservoirs are Markovian is only an approximation in most systems. The above comparison is only to put our results in context with these predictions and not to verify them in our engine. In conclusion, all bounds on efficiency and predictions on efficiency at maximum power were made on thermal engines. All these predictions match with our observations for $\tau_{\text{cycle}} > \tau_{\text{h}}$, where both isotherms and isochores are at least partially equilibrated. For thermal engines, both isotherms and isochores should fail at τ_{h} and had this been the case with our engine, these predictions might as well have been true for all cycle times and power-efficiency tradeoff might still be true. Selective engineering of the relaxation times using non-Markovian noise created a unique regime in our experiment where the tradeoff gets reversed and all theoretical predictions fail exactly when the relaxation times differ from that of thermal engines.

Supplementary Note 8: Ultimate fate of the heat engine and inspiration to engineering the regime of operation

In Fig. 4 in the main paper, $\tau_{\text{cycle}} = 12$ ms was the lowest cycle time explored in our experiments. As determined in Fig. 3d of the main paper, isochoric process starts to fail if an engine was operated at $\tau_{\text{cycle}} < \tau_{\text{R}} = 12$ ms, and T_{eff} would not increase(decrease) during the isochoric heating(cooling). Operating an engine under such circumstances would be equivalent to performing a fast Stirling cycle at constant T_{eff}. To explore this ultimate

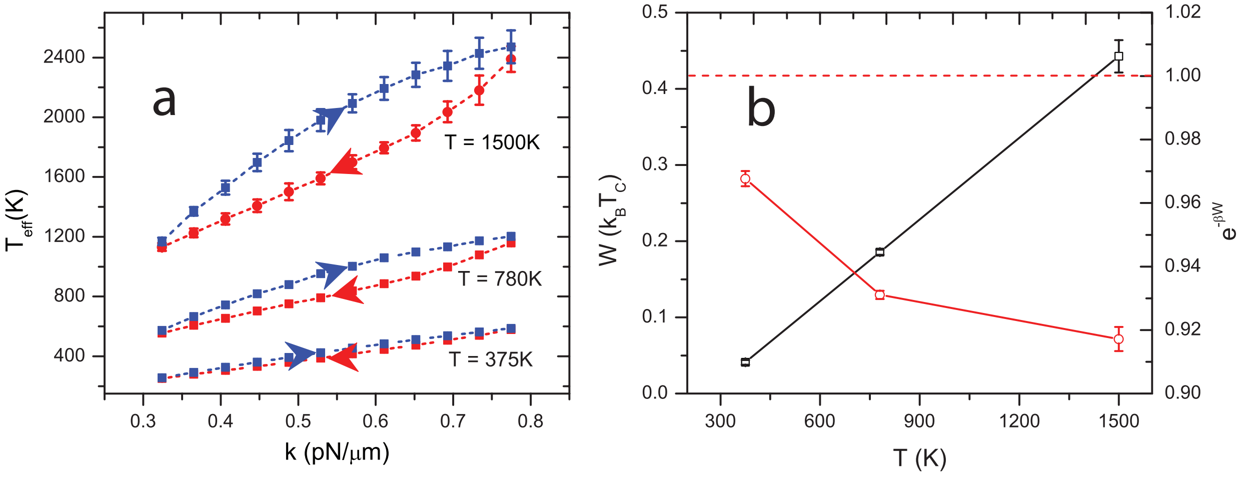

Supplementary Figure 10. **Ultimate fate of the system and Jarzynski inequality at low τ.** **a** shows trajectories of the system in the $k - T_{\text{eff}}$ plane when only the k protocol is performed at various T_H. The dotted lines represent the progression of each cycle with the arrow indicating its sense. The black open squares in **b** represent the W in these, while the red open circles denote the RHS of Jarzynski inequality. Averaging and error bars are the same as Fig. 5d and is presented in Supplementary Note 4

fate of the engine, we performed the k protocol in Fig. 3a of the main paper at constant T_{eff} = 375K, 780K and 1500K for $\tau_{\text{cycle}} = 15$ ms and traced the state of the system in the $k - T_{\text{eff}}$ plane in Supplementary Figure 10a. With the path taken by the system during compression above that of the expansion for all T_{eff}, work is done by the surrounding on the system i.e. $W > 0$ as can be seen in Supplementary Figure 10a. Thus, at very low $\tau_{\text{cycle}} < 12$ ms, η eventually decreases as $\tau_{\text{cycle}} \to 0$.

The results in Supplementary Figure 10a represent the most common mechanism in which macroscopic engines fail to overcome $P - \eta$ tradeoff. In all macroscopic engines, design constraints result in failures in isochoric processes to occur much faster than the isotherms [23, 24]. A natural question arising from these observations was 'What if we could reverse the scenario and let the isotherms fail faster than the isochores?'. Pursuing such an endeavor, however, has not been possible with macro-engines. Nonetheless, manipulating engine-bath interactions and tuning the relaxation times is relatively simple in micro-engines and this possibility lead us to the regime of operation explored in our paper. An intuitive guess for the outcome of such a change would be that it would push the hot isotherm above

the cold isotherm as equilibration during the isochores would now occur much faster. This was indeed observed in our experiments and as demonstrated in Fig. 5 of the main paper, reducing τ_{R} during the isochores pushed the T_{eff} in the hot isotherm above the cold isotherm.

From Jarzynski's inequality, we have $e^{-\beta\Delta F} \geq e^{-\beta W}$, where ΔF and W are change in free energy and work done during a process. $\beta = \frac{1}{k_{\mathrm{B}}T}$, where T is the temperature of the system and k_{B}, the Boltzmann constant. $\Delta F = 0$ over the full period of a cyclic process and L.H.S of the inequality is 1. The R.H.S is plotted in Supplementary Figure 10b and is less than 1 for all T_{eff} used in the experiment. Thus, Jarzynski's inequality holds true for our system even at low τ.

Supplementary Note 9: Heat engine at higher temperature

In Fig. 5 of the main paper, we discussed that operating at a $\tau_{\mathrm{cycle}} < \tau_h$ resulted in a failure of volume equilibration and T_{eff} increased (decreased) linearly along the cold (hot) isotherm. Intuitively, instead of the isochoric heating where heat was drawn from the reservoir, internal energy of the particle is now used in heating the engine by compressing quickly. The change in volume (k_{max} & k_{min}) determines the maximum temperature, T_h ($\approx 560K$ in Fig 4) that can be reached by such a compression. In Figure 4, T_{max} is less than T_{H} and the isochoric process was not required.

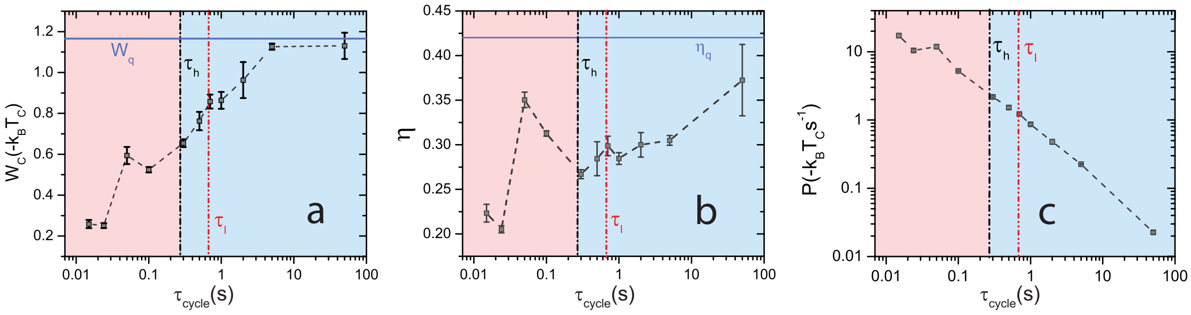

Supplementary Figure 11. **Breakdown of the reversal of $P - \eta$ tradeoff at high $T_{\mathbf{max}} = 935K$. a-c** show W_{C} (Squares), η (Circles) and P (Diamonds) are shown for τ_{cycle} spanning over two decades above and below the relaxation times τ_{h} (black dash dotted line) and τ_{l}(red dash dot dot line) in **a**, **b** and **c** respectively. The quasi-static limits W_{q} and η_{q} defined by equilibrium thermodynamics are represented as blue solid lines in **a** and **b**.Averaging and error bars are the same as Fig. 4 and is presented in Supplementary Note 4

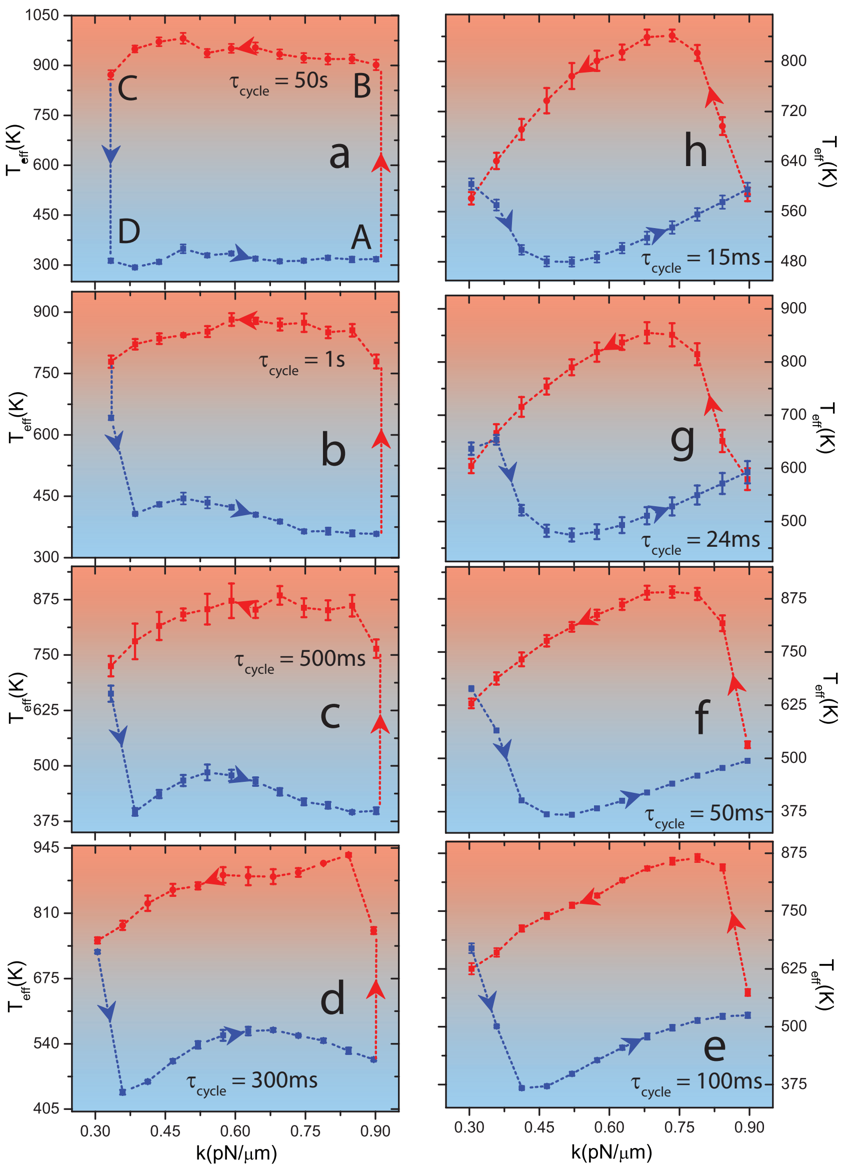

Supplementary Figure 12. **$k - T_{\text{eff}}$ diagrams for Stirling cycle with T_{eff} = 935K. a-h** represent the state of the system on the $k - T_{\text{eff}}$ plane for τ_{cycle} = 50s, 1s, 500ms, 300ms, 100ms, 50ms, 24ms, 15ms respectively, where, the red circles are state points in contact with hot reservoir(T_h) and blue squares with cold reservoir(T_l). The trajectory for τ_{cycle} = 50s was averaged over 70 cycles, τ_{cycle} = 1s over 2700, τ_{cycle} = 500 ms over 5400, τ_{cycle} = 300 ms over 9000, τ_{cycle} = 100 ms over 27000, τ_{cycle} = 50 ms over 54000, τ_{cycle} = 24 ms over 100000 and τ_{cycle} = 300 ms over 180000 cycles respectively. The dotted line are a guide to the eye with the arrows denoting the direction of progress of Stirling cycle. Averaging and error bars are the same as Fig. 5 and is presented in Supplementary Note 4

In Supplementary Figure 11, we explore an engine, where, $T_{\text{max}} > T_{\text{H}}$. To determine, T_{H}, we assumed that all the internal energy at the start of compression gets converted to increase in temperature, which, in our experiments is $\approx 8000K$. The results in Supplementary Figure 11 represent the performance of an engine with $T_{\text{max}} = 935K$. As earlier, for $\tau > \tau_{\text{h}}$, W_{C} and η decrease as $\tau \to \tau_{\text{h}}$. At $\tau < \tau_{\text{h}}$, after a brief saturation in W_{C} and an increase in η, both W_{C} and η decrease as $\tau \to 0$. To understand this breakdown in the upturn in η at higher T_{max}, we plot the state of the system in $k - T_{\text{eff}}$ plane in Supplementary Figure 12. At large τ, our observations matched with that in the quasistatic limit and the trajectory formed a rectangle ABCD in the $k - T_{\text{eff}}$ plane as shown in Supplementary Figure 12a. At $\tau = 300$ ms, since $T_{\text{max}} > T_{\text{H}}$, the path in the $k - T_{\text{eff}}$ plane now appears as a distorted rectangle (Supplementary Figure 12d), where, due to insufficient internal energy, part of heat transferred by the hot reservoir, Q_{h} is still used in heating the system. However, on faster cycling at $\tau < \tau_{\text{h}}$, the cooling down during $B \to C$ occurs even before T_{max} is reached as shown in Supplementary Figure 12e, enabling T_{eff} at C to decrease further. The part of Q_h used in heating the engine is the difference in internal energy(temperature) between A and C (250K in Supplementary Figure 12d and 100K in Supplementary Figure 12e), now decreases to briefly saturate W_{C} and increase η. Similarly, as $\tau \to 0$, the heating while $D \to A$ starts even before the system cools down to T_{min} (at 480K in Supplementary Figure 12f), thus, reducing W_{C} and η. The reversal of $P - \eta$ tradeoff demonstrated in Fig. 4, hence, occurs for $T_{\text{max}} < T_{\text{H}}$ set by the bounds of operation k_{max} and k_{min}. τ_{l} and τ_{h}, also dependent only on k_{max} and k_{min}, remain the same across all T_{max} and decide the turnover in $P - \eta$ curves. On the other hand, τ_{R} during the isochores increases with T_{max}, suggesting an even earlier failure in the reversal of $P - \eta$ tradeoff for higher temperatures.

To further explore the temperature dependence of the reversal in $P - \eta$ tradeoff, we performed similar experiments at an even higher temperature $T_{\text{eff}} = 1310$K. As earlier, τ_{h} and τ_{l} remain the same, but τ_{R} for isochoric process is now significantly higher. W_{C}, η and P for these are plotted in Supplementary Figure 13. As in the case of $T_{\text{eff}} = 935$K, the upturn in η breaks down at higher temperatures. At very low $\tau = 15$ ms, however, there is a slight reversal.

To understand the performance of the engine, we perform the same drill as before and analyzed the trajectories of the system in the $k - T_{\text{eff}}$ plane in Supplementary Figure 14. As in Supplementary Figure 12, due to a larger τ_{R}, cooling the system back to T_{min} now

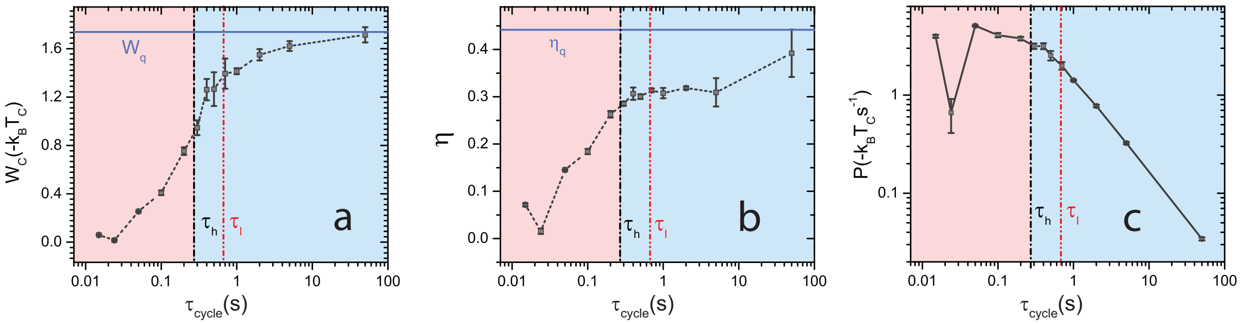

Supplementary Figure 13. **Breakdown of the reversal of $P-\eta$ tradeoff at very high $T_{\mathbf{max}}$ = 1310K. a-c** show W_{C} (Squares), η (Circles) and P (Diamonds) are shown for τ_{cycle} spanning over two decades above and below the relaxation times τ_{h} (black dash dotted line) and τ_{l}(red dash dot dot line) in **a**, **b** and **c** respectively. The quasi-static limits W_{q} and η_{q} defined by equilibrium thermodynamics are represented as blue solid lines in **a** and **b**. Averaging and error bars are the same as Fig. 4 and is presented in Supplementary Note 4

takes considerable time even at τ_{cycle} as high as $1s$. At 500 ms, even at the end of the cold cycle, the system does not reach T_C and the engine heats up as observed in macroscopic systems. The inability to explore the boundaries of the system starts to take effect below $\tau_{\mathrm{cycle}} = 100$ ms and T_{eff} of the hot isotherm now falls at k_{min}, while that of the cold isotherm rises at k_{max}. Although, the internal energy difference across the hot cycle decreases as in Supplementary Figure 12, it is insufficient to increase η significantly. Ultimately, at very low $\tau = 15$ ms, the system does reach a state similar to Fig. 5d of the main paper, albeit with a much smaller area in comparison with $\tau = 50s$, thus, slightly increasing W_{C} and η.

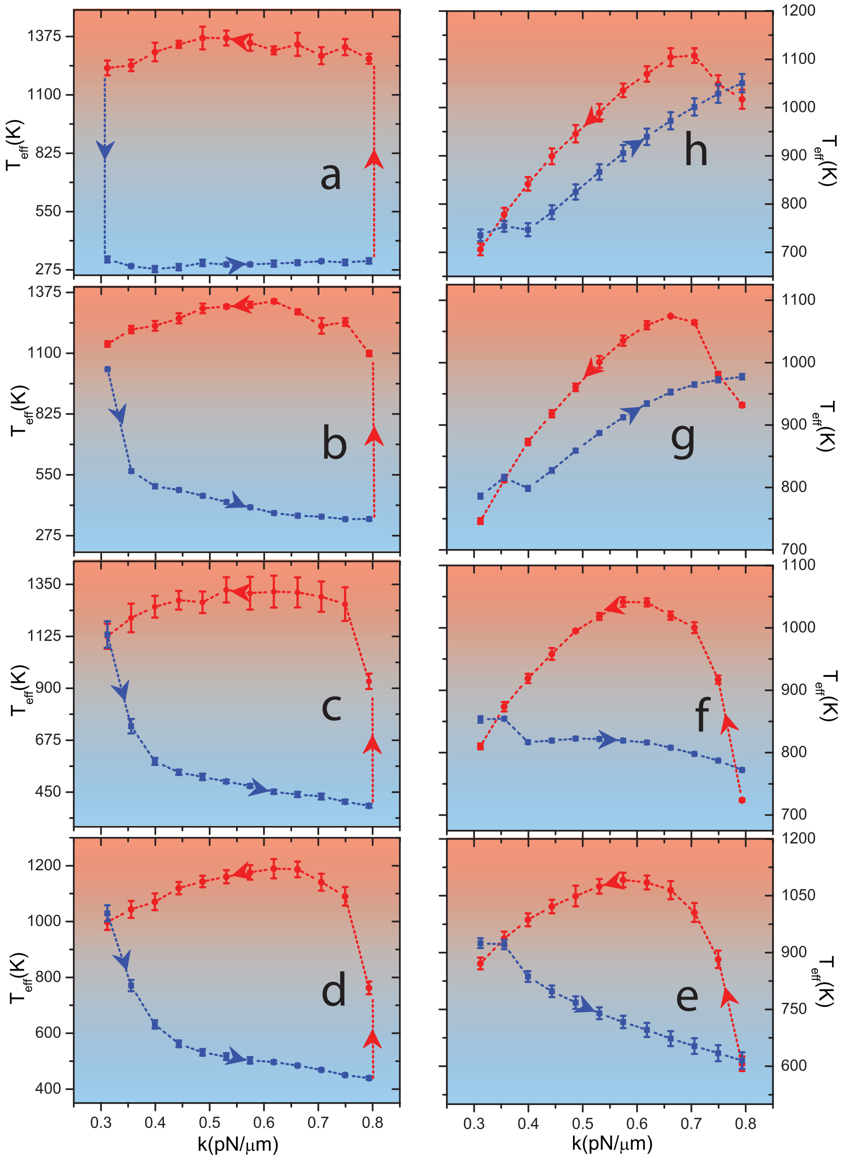

Supplementary Figure 14. $k - T_{\text{eff}}$ **diagrams for Stirling cycle with** T_{eff} **= 1310K. a-h** represent the state of the system on the $k - T_{\text{eff}}$ plane for τ_{cycle} = 50s, 1s, 500ms, 300ms, 100ms, 50ms, 24ms, 15 ms respectively, where, the red circles are state points in contact with hot reservoir(T_{max}) and blue squares with cold reservoir(T_{min}). The trajectory for $\tau_{\text{cycle}} = 50s$ was averaged over 70 cycles, $\tau_{\text{cycle}} = 1s$ over 2700, τ_{cycle} = 500 ms over 5400, τ_{cycle} = 300 ms over 9000, τ_{cycle} = 100 ms over 27000, τ_{cycle} = 50 ms over 54000, τ_{cycle} = 24 ms over 100000 and τ_{cycle} = 300 ms over 180000 cycles respectively. The dotted line are a guide to the eye with the arrows denoting the direction of progress of Stirling cycle. Averaging and error bars are the same as Fig. 5 and is presented in Supplementary Note 4

[1] Grosse, Constantino, & Alejandra Veronica Delgado. Dielectric dispersion in aqueous colloidal systems. *Curr. Opin. Colloid Interface Sci.*. **15**, 3:145-159 (2010).

[2] Squires, T. M., & Quake, S. R. Microfluidics: Fluid physics at the nanoliter scale. *Rev. Mod. Phys*, **77**, 3 (2005).

[3] Squires, T. M., & Bazant, M. Z. Induced-charge electro-osmosis. *J. Fluid Mech*, **509**, 217-252 (2004).

[4] Ahualli, S., A. Delgado, S. J. Miklavcic, & L. R. White. Dynamic electrophoretic mobility of concentrated dispersions of spherical colloidal particles. On the consistent use of the cell model. *Langmuir* **22**, 16: 7041-7051 (2006).

[5] Semenov, Ilya, et al. Single colloid electrophoresis. *Curr. Opin. Colloid Interface Sci.* **337**.1: 260-264 (2009).

[6] Bazant, M. Z., Thornton, K. & Ajdari, A. Diffuse-charge dynamics in electrochemical systems. *Phys. Rev. E* **70**, 021506 (2004)

[7] Pesce, Giuseppe, Vincenzo Lisbino, Giulia Rusciano, & Antonio Sasso. Optical manipulation of charged microparticles in polar fluids. *Electrophoresis* **34**, 22-23: 3141-3149 (2013).

[8] Otto, Oliver, Christof Gutsche, Friedrich Kremer, & Ulrich F. Keyser. Optical tweezers with 2.5 kHz bandwidth video detection for single-colloid electrophoresis. *Rev. Sci. Instr.* **79**, 2 (2008).

[9] Roldán, Édgar, Ignacio A. Martínez, Luis Dinis, & Raúl A. Rica. Measuring kinetic energy changes in the mesoscale with low acquisition rates. *App. Phys. Lett.* **104**, 23 (2014).

[10] Mestres, Pau, Ignacio A. Martinez, Antonio Ortiz-Ambriz, Raul A. Rica, & Edgar Roldan. Realization of nonequilibrium thermodynamic processes using external colored noise. *Phys. Rev. E* **90**, 3: 032116 (2014).

[11] Alireza Seif, Sarah A.M. Loos, Gennaro Tucci, Edgar Roldan & Sebastian Goldt. The impact of memory on learning sequence-to-sequence tasks. arXiv:2205.14683 (2023).

[12] Sekimoto, K. Langevin equation and thermodynamics. Prog. Theor. Phys. Suppl. 130, 17–27 (1998).

[13] Seifert, U. Stochastic thermodynamics, fluctuation theorems and molecular machines. *Rep. Prog. Phys.* **75**, 126001 (2012).

[14] Blickle, V., Speck, T., Helden, L., Seifert, U. & Bechinger, C. Thermodynamics of a colloidal particle in a time-dependent nonharmonic potential. *Phys. Rev. Lett.* 96, 070603 (2006)

[15] Martinez, I. A. et.al. Brownian Carnot engine. *Nat. Phys.* **12**, 67-70(2016)

[16] Arya Datta, Patrick Pietzonka, & Andre C. Barato. Second Law for Active Heat Engines. *Phys. Rev. X.* **12**, 031034 (2022).

[17] Chupeau, M., Besga, B., Guery-Odelin, D., Trizac, E., Petrosyan, A., & Ciliberto, S. Thermal bath engineering for swift equilibration. *Phys. Rev. E*, **98**(1), 010104, (2018).

[18] Niloyendu Roy, Nathan Leroux, AK Sood & Rajesh Ganapathy. Tuning the performance of a micrometer-sized Stirling engine through reservoir engineering. *Nat. Commun.*, **12**, 4927, (2021).

[19] Curzon, F. L. & Ahlborn, B. Efficiency of a Carnot engine at maximum power output. *Am. J. Phys.* **43**, 22-24 (1975).

[20] Schmiedl, T. & Seifert, U. Efficiency at maximum power: an analytically solvable model for stochastic heat engines. *Euro. Phys. Lett.* **81**, 20003(2008)

[21] Pietzonka, P., & Seifert, U. Universal trade-off between power, efficiency, and constancy in steady-state heat engines. *Phys. Rev. Lett.*, **120**(19), 190602, (2018).

[22] Koyuk, T. & Seifert, U. Operationally Accessible Bounds on Fluctuations and Entropy Production in Periodically Driven Systems. *Phys. Rev. Lett.*, **122**, 230601 (2019)

[23] Egas, J., & Don M. Clucas. Stirling engine configuration selection. *Energies* **11**, 3: 584, (2018).

[24] Damirchi, H., et.al. . Design, fabrication and evaluation of gamma-type stirling engine to produce electricity from biomass for the micro-CHP system. *Energy Procedia* **75**:137-143, (2015).
